# Self-powered cathodic detection of dissolved oxygen using a paper-based biofuel cell

**DOI:** 10.1039/d5ra09344a

**Published:** 2026-02-02

**Authors:** Isao Shitanda, Riko Ohkura, Noya Loew, Hikari Watanabe, Seiya Tsujimura, Masayuki Itagaki

**Affiliations:** a Department of Pure and Applied Chemistry, Faculty of Science and Technology, Tokyo University of Science Noda 2641 Yamazaki Chiba 278-8510 Japan shitanda@rs.tus.ac.jp; b Research Institute for Science and Technology, Tokyo University of Science 2641 Yamazaki Noda Chiba 278-8510 Japan; c Division of Materials Sciences, Faculty of Pure and Applied Sciences, University of Tsukuba 1-1-1 Tennodai Tsukuba Ibaraki 305-8573 Japan

## Abstract

Herein we report the self-powered biosensor for detection of dissolved oxygen (DO) detection using a paper-based enzymatic biofuel cell (BFC) employing screen-printed electrodes composed of MgO-templated mesoporous carbon (MgOC). The sensor used an anode modified by flavin adenine dinucleotide-dependent glucose dehydrogenase (FAD-GDH) and a cathode modified by bilirubin oxidase (BOD) to enable selective oxygen reduction under glucose-rich conditions. Electrochemical analyses revealed a linear relationship between the cathodic current and DO concentration over the range of 0–22 mg L^−1^, with a maximum power output of 398 µW cm^−2^ at 20 mg L^−1^ DO. The biosensor system was successfully used to quantify DO in both pure water and a commercial soft drink, without requiring external power sources. These findings demonstrate the feasibility of low-cost, disposable, and scalable DO sensing by using cathode-targeting enzymatic BFCs, thereby opening new avenues for environmental and food quality monitoring.

## Abstract 2

Herein we report the self-powered biosensor for detection of dissolved oxygen (DO) detection using a paper-based enzymatic biofuel cell (BFC) employing screen-printed electrodes composed of MgO-templated mesoporous carbon (MgOC).

## Graphical abstract

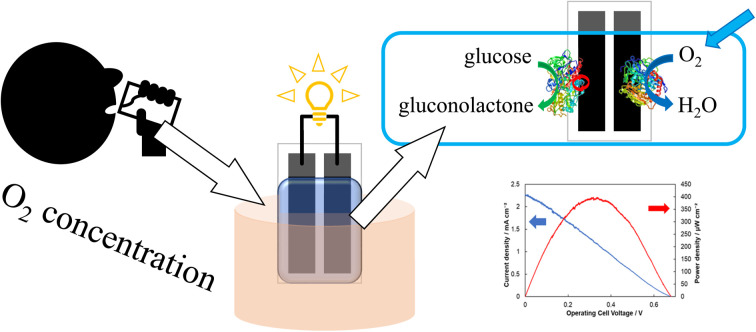

## Introduction

Monitoring dissolved oxygen (DO), *i.e.*, the molecular oxygen dissolved in liquids, is critical for a wide range of applications, including the environmental assessment of aquatic systems, food safety, aquaculture, clinical diagnostics, and biotechnological processes like microbial and tissue cultures. Traditional DO detection techniques include fluorescence quenching,^1,2^ Winkler titration,^2–4^ and the use of electrochemical sensors like Clark electrodes.^5–7^ However, these methods typically require external power sources, or as in the case of titration, technically skilled operators, limiting their suitability for rapid, low-cost, and on-site measurements.

Self-powered sensors, which operate without external energy sources, have emerged as promising tools for portable and low-resource diagnostics.^8,9^ Among these, biofuel cells (BFCs) function as enzyme-based self-powered biosensors under fuel-limited conditions. BFCs are fuel cells that employ biological components, such as an enzymes, organelles, or whole cells, to break down the fuel and extract energy in form of electricity.^10–13^ These BFCs can generate electrical signals correlated with analyte concentration. While most reported BFC-based biosensors target anodic fuels, such as glucose or lactate,^14–18^ there have been few studies exploring their use in detecting cathodic fuels, such as molecular oxygen. However, theoretical oxygen quantification based on the cathodic reaction in the presence of excess anodic fuel is an overlooked sensing strategy.

In our previous study, we developed high-performance paper-based BFCs using screen-printed electrodes, offering low-cost, scalable, and disposable platforms for biosensing applications.^16–20^ The combination of screen-printing and the use of paper substrates enables facile mass fabrication and effective fuel distribution by the intrinsic wicking effect, enhancing device performance. Prior studies have demonstrated notable power outputs using such platforms, including 37.5 and 46.4 µW cm^−2^ using 5 and 30 mM glucose, respectively.^21,22^ Building on this, our group achieved 120 µW cm^−2^ with 25 mM glucose and enabled wireless signal transmission for real-time sensing.^18^ Central to this advancement was the use of MgO-templated mesoporous carbon (MgOC) electrodes, which offer tunable pore sizes, high surface areas, and excellent biocompatibility, which are critical for efficient enzyme immobilization and electron transfer.^23–32^

In this study, we extended our past research to develop a self-powered oxygen biosensor. We achieved selective and quantitative detection of dissolved oxygen, enabled by cathodic current generation using MgOC-based paper electrodes modified by bilirubin oxidase and glucose dehydrogenase. The results of this study underscored the potential of cathodic-targeting BFCs as practical and disposable sensors for environmental and biomedical applications.

## Experimental section

### Materials and enzyme preparation

The porous carbon ink was prepared by following a previously reported method.^16^ Specifically, 0.75 g of MgO-templated carbon (MgOC; CNovel©, Toyo Tanso Co. Ltd; pore size: 72 nm) was mixed with 2.5 mL of 1-methyl-2-pyrrolidone (NMP) and 7.5 mL of polyvinylidene fluoride (PVdF; #9305; 5% in NMP; Kureha Corporation) using a rotation–revolution mixer (ARE-310, Thinky, Japan) for 1 min. The enzymes flavin adenine dinucleotide-dependent glucose dehydrogenase (FAD-GDH) and bilirubin oxidase (BOD) were obtained from Amano Enzyme (Japan). All reagents were of analytical grade.

### Electrode fabrication

Japanese paper (Kagesen Izumo, Keynote Planning), which was employed as the substrate, was first rendered water-repellent using a commercial coating agent (Hajikkusu, Komensu, Japan). A current collector was then screen-printed in five layers onto the substrate using carbon paste (JELCON CH-10, Jujo Chemical), and each layer was cured at 120 °C for 30 min in an electric oven. To enhance the oxygen supply, the cathode lead pattern incorporated 25 holes, each 1 mm in diameter, based on a previous design.^16^ These holes in the cathode lead allow oxygen to diffuse through the paper substrate directly into the porous carbon layer, bypassing the dense carbon lead.^16^ Two layers of porous carbon ink (5 mm × 20 mm) were then printed onto the current collector, and each layer was dried at 60 °C for 24 h.

### Enzyme modification

To modify the anode, 20 µL of 250 mM 1,2-naphthoquinone (1,2-NQ) solution in acetonitrile was dropped onto the porous carbon layer and dried for 15 min. Subsequently, the FAD-GDH solution (20 U µL^−1^; 20 µL per electrode) dissolved in 10 mM phosphate buffer (pH 7.0) was cast and dried under reduced pressure for 1.5 h. To modify the cathode, BOD (1 U µL^−1^; 20 µL per electrode) in a 10 mM phosphate buffer (pH 7.0) was applied and dried for 1 h under reduced pressure.

### Electrochemical measurements

**Fig. 1:**
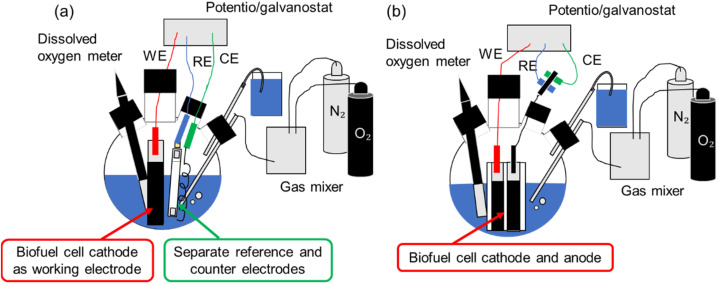
Schematic of the electrochemical measurement setup of (a) a single electrode and (b) biofuel cell.

The electrochemical evaluations were conducted in a flask cell containing a 1 M phosphate buffer solution as the electrolyte (Fig. 1). A potentiostat/galvanostat (EmStat3, PalmSens, Netherlands) was used for measurements. For measurements involving the biocathode, the solution was either air-saturated or contained a controlled DO in the concentration range of 0–22 mg L^−1^. To control the DO, the system was sealed and equipped with a DO meter and a gas flow controller. For measurements involving the bioanode, the solution contained glucose. To characterize the biocathode and bioanode individually, measurements were performed using a three-electrode setup with the enzyme-modified paper electrodes as the working electrodes, with a platinum wire and saturated KCl Ag/AgCl electrode functioning as the counter and reference electrodes, respectively (Fig. 1a). BFC measurements were performed using a two-electrode setup with the paper-based biocathode and the paper-based bioanode as the two electrodes (Fig. 1b). Cyclic voltammetry (CV) was performed at a scan rate of 10 mV s^−1^. Chronoamperometry (CA) was conducted at 0.3 V for 300 s. Linear sweep voltammetry (LSV) was carried out at a scan rate of 1 mV s^−1^.

### Real sample measurements

To simulate real-world applications, LSV was performed using pure water and a commercially available soft drink (Otsuka Pharmaceutical Co., Japan) as the test samples. For measurements in glucose-free solutions, a 1 M phosphate buffer containing 200 mM gelatinized glucose was applied to the paper electrode and covered with a porous film to prevent mixing. The system was sealed and operated under the same electrochemical protocol described above. The soft drink was measured as-is and immediately after opening.

## Results and discussion

### Electrochemical characterization of electrodes

The CV results of the anode and cathode are shown in Fig. S1. The anode exhibits a higher catalytic current in the presence of glucose, indicating that glucose oxidation is effectively mediated by FAD-GDH. At the cathode, the catalytic current increased significantly under oxygen-saturated conditions, confirming that BOD efficiently catalyzes oxygen reduction.

### Cathodic response to dissolved oxygen concentration

**Fig. 2:**
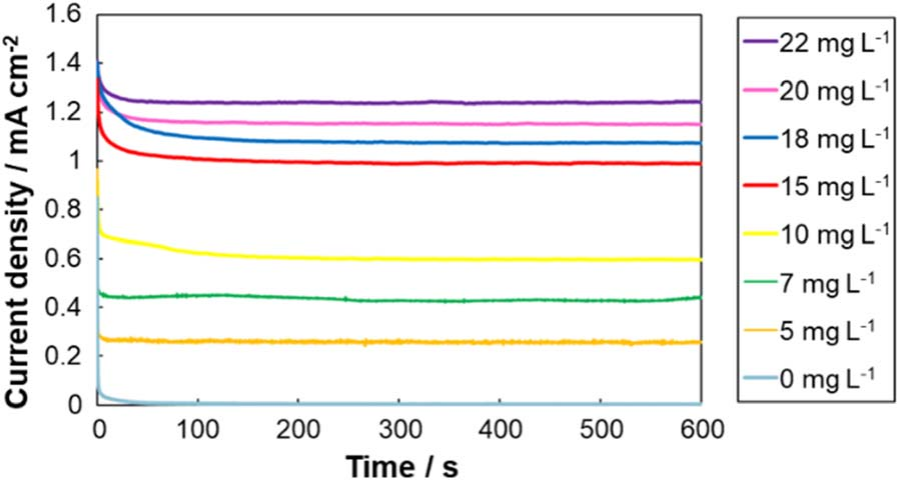
Chronoamperometry (CA) responses of BOD-modified cathode in the presence of dissolved oxygen at different concentrations (0–22 mg L^−1^).

**Fig. 3:**
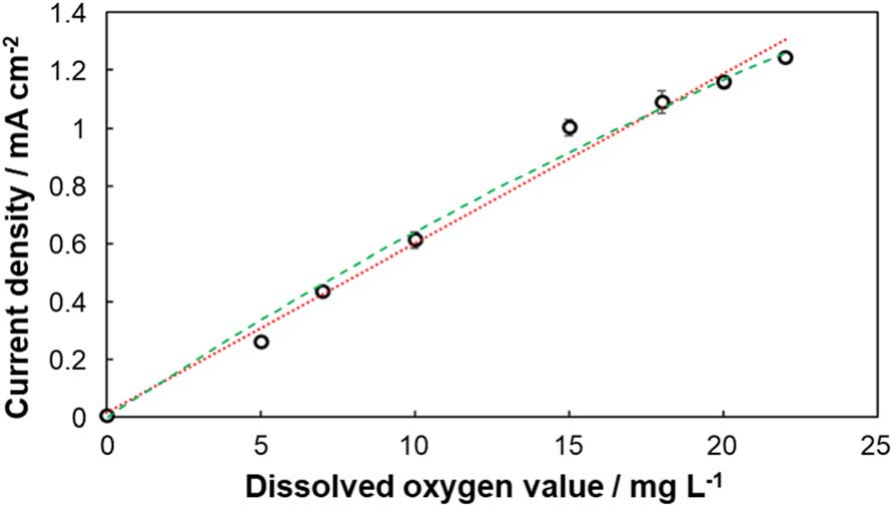
Calibration curves of BOD-modified cathode in the presence of dissolved oxygen created from the results of CA at different concentrations (0–22 mg L^−1^). Red dotted line (linear) sensitivity: 0.057 mA cm^−2^ mg^−1^ L, *R*^2^ = 0.984; Green dashed line (Michaelis–Menten) *K*_m_ = 90.4 mg L^−1^, *I*_max_ = 6.45 mA cm^−2^, *R*^2^ = 0.996. Error bars indicate 90% confidence interval; *N* = 3.

CA measurements were conducted to investigate the cathodic response in the presence of dissolved oxygen (DO) at different concentrations. As illustrated in Fig. 2, the reduction current at the BOD-modified cathode increased proportionally as DO concentration increased from 0 to 22 mg L^−1^. This linear correlation enabled the construction of a calibration curve (Fig. 3), demonstrating the feasibility of using the cathodic system for quantitative DO sensing. As a comparison, a Michaelis–Menten-type fit is also shown.

### BFC output performance at different DO levels

**Fig. 4:**
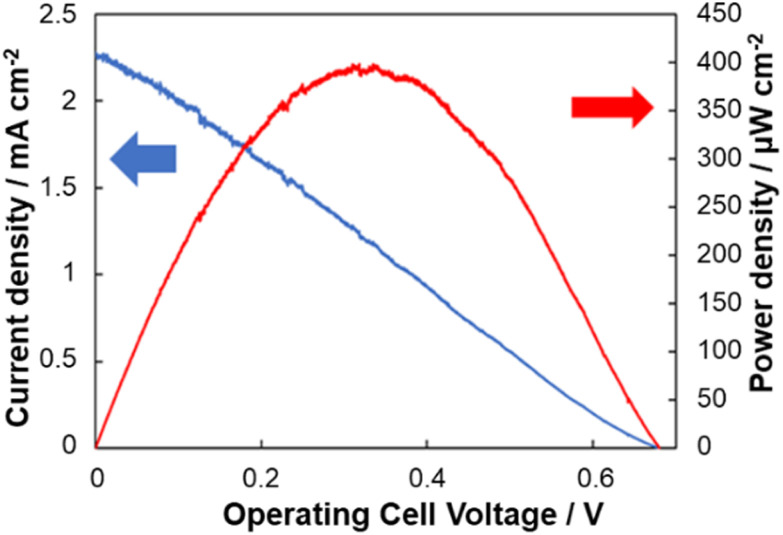
Voltage-output curves and voltage–current curves of biofuel cells using paper-based electrodes in 1 M phosphate buffer containing 200 mM glucose and DO levels of 20 mg L^−1^.

**Fig. 5:**
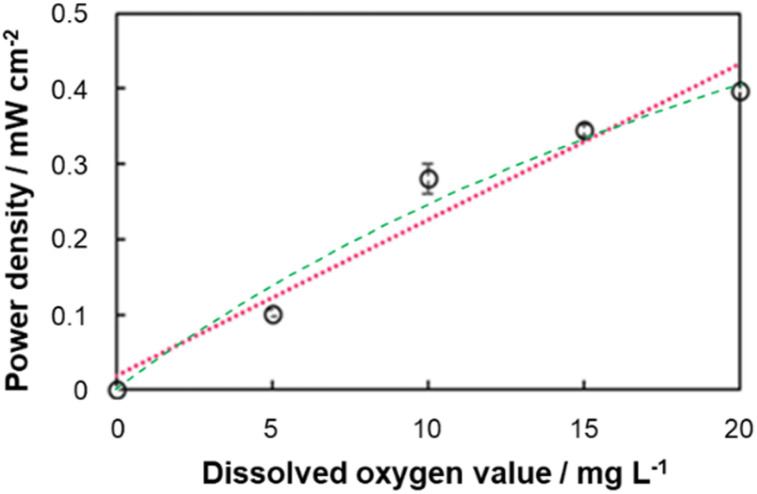
Calibration curves of biofuel cells using paper-based electrodes created from power output in 1 M phosphate buffer containing 200 mM glucose and DO levels of 0–20 mg L^−1^. Red dotted line (linear) sensitivity: 0.021 mW cm^−2^ mg^−1^ L; *R*^2^ = 0.953; Green dashed line (Michaelis–Menten) *K*_m_ = 37.0 mg L^−1^, *P*_max_ = 1.17 mW cm^−2^, *R*^2^ = 0.976. Error bars indicate 90% confidence interval; *N* = 3.

LSV was used to assess the power output of the BFC for different DO concentrations. Fig. 4 shows the BFC performances for DO levels of 20 mg L^−1^. The maximum power densities recorded were 1.40 µW cm^−2^ (0 mg L^−1^), 96.5 µW cm^−2^ (5 mg L^−1^), 280 µW cm^−2^ (10 mg L^−1^), 345 µW cm^−2^ (15 mg L^−1^), and 398 µW cm^−2^ (20 mg L^−1^). Both linear and Michaelis–Menten type calibration curves are shown in Fig. 5. The linear calibration was used for DO quantification in real samples. Application examples for this self-powered sensor include quick and simple spot-checking the DO of drinks and other water samples to help decide if further action is needed. The accuracy of the sensor as shown in Fig. 5 is sufficient for such applications.

### Performance in pure water

**Fig. 6:**
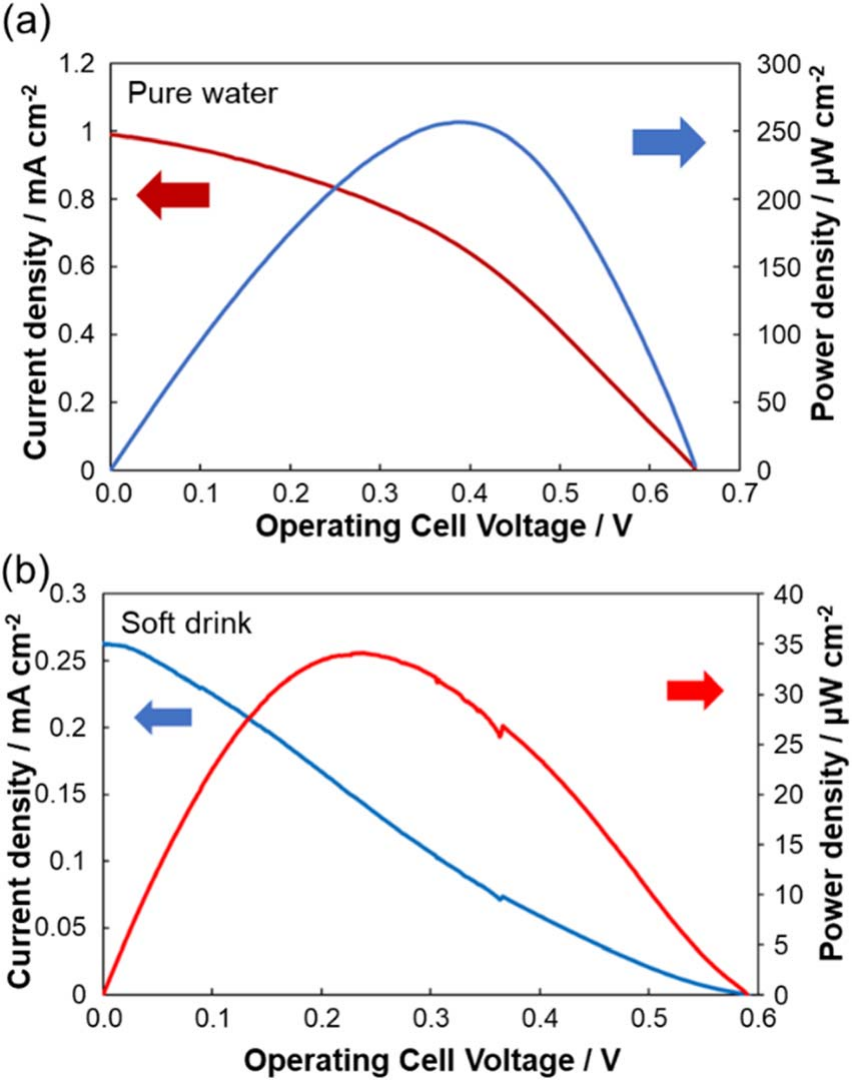
Voltage-output curves and voltage–current curves of biofuel cells using paper-based electrodes for pure water to estimate DO concentration (a) and soft drink samples showing reduced DO concentration (b).

A common approach to enable biosensor operation in samples lacking critical reagents is to provide those reagents as a component of the sensing device. In this study, a gelled glucose-containing phosphate buffer was applied to the electrode to evaluate the functionality of the sensor in glucose-free test solutions. This configuration allowed the measurement of DO in pure water while maintaining anodic activity. The sensor generated an open-circuit voltage (EMF) of 0.65 V, a maximum current density of 0.99 mA cm^−2^, and a maximum power density of 257 µW cm^−2^ (Fig. 6a). From the calibration curve (Fig. 5), the DO concentration in pure water at 28 °C was estimated to be 11.5 mg L^−1^, which was consistent with values obtained using any commercial DO meter.

### Application to real sample (soft drink)

The sensor was further evaluated using a commercial soft drink as a test sample to demonstrate its applicability in complex matrices. As shown in Fig. 6b, the system generated a current density of 0.263 mA cm^−2^, an EMF of 0.589 V, and a maximum power density of 34.1 µW cm^−2^. The corresponding DO concentration, estimated using the calibration curve, was approximately 0.77 mg L^−1^, which agreed with expected DO levels in carbonated beverages obtained immediately after opening.

## Discussion

While the biosensing system in this study was operated using a potentiostat, the results demonstrate that paper-based BFCs can function as self-powered biosensors for DO measurement. Because the output power is DO level-dependent, connecting the BFCs developed here to a power-quantifying device will result in a full self-powered biosensor system. The error bars in Fig. 3 and 5 indicate high reproducibility of the electrodes, and the linear range shown in these figures covers the typical concentration range of oxygen in water. Furthermore, while the Michaelis–Menten-type calibration is slightly more accurate, as is common with enzyme-based electrochemical systems, the accuracy of the linear calibration is sufficient for most practical applications. Therefore, the simpler linear calibration was preferred in this study. However, a Michaelis–Menten-type calibration is possible, should it be needed. It should be noted that the apparent *K*_m_ value of the BFC calibration (Fig. 5) is significantly lower than the *K*_m_ value of biocathode calibration (Fig. 3). As enzyme-based electrodes, the outputs of both the biocathode and the bioanode are expected to saturate at high DO and glucose concentrations, respectively. The lower *K*_m_ value of the BFC calibration indicates that the maximal output of the bioanode is lower than that of the biocathode, so that the total outut power of the BFC saturates at a lower DO concentration. Consequently, improving the performance of the bioanode should lead to an improved accuracy and range of the self-powered biosensor.

The linear sensitivity of the biofuel cell was adequate for DO sensing, at 0.021 mW cm^−2^ mg^−1^ L DO (Fig. 5). Finally, the measured DO concentration of the soft drink was consistent with the expected DO level, indicating that sugars and other components in the beverage do not significantly influence the sensor output. Particularly, glucose is unlikely to influence sensor response, as the anode performance is maximized at the glucose concentration incorporated into the gel. Further studies are needed to confirm these findings.

The stability of the self-powered DO sensor is expected to be similar to that of corresponding self-powered sensors for anodic fuels.^18,33^ Additional studies are needed to confirm. If necessary, similar methods as for anodic fuel sensors can be utilized to enhance stability. Furthermore, the amount of glucose in the gelled buffer might need to be increased for extended operations. In other words, the self-powered DO sensor is suitable for short-term or controlled applications without further testing. The reliability in long-term applications or demanding environments cannot be fully guaranteed without further testing.

## Conclusions

In this study, we developed a self-powered biosensor for dissolved oxygen (DO) detection using paper-based biofuel cells (BFCs) employing MgO-templated mesoporous carbon (MgOC) screen-printed electrodes. The results confirm that the cathodic current and power output of the self-powered paper-based BFC biosensor are directly proportional to the DO concentration in the electrolyte. This enabled accurate quantification in both pure water and complex real sample matrices. The system demonstrated sufficient sensitivity, linearity, reliability, and operational simplicity without requiring any external power source, highlighting the potential of cathode-targeting BFCs as practical, disposable, and low-cost sensors for environmental and food analyses. Future research will be focused on integrating the sensor with wireless systems for real-time DO monitoring, and expanding its application to other redox-active analytes.

## Author contributions

I. S. and S. T. conceptualized the work. R. O. carried out the experiments. I. S. and N. L. wrote the manuscript. I. S., H. W., S. T. and M. I. supervised the work.

## Conflicts of interest

There are no conflicts to declare.

## Supplementary Material

RA-016-D5RA09344A-s001

## Data Availability

All data supporting the findings of this study are included within the supplementary information (SI). Supplementary information is available. See DOI: https://doi.org/10.1039/d5ra09344a.
