# Supplementary material for: Self-powered cathodic detection of dissolved oxygen using a paper-based biofuel cell
Source: RSC Adv. 2026 Feb 2;16(7):6502–6. doi: 10.1039/d5ra09344a (PMC12862783; doi:10.1039/d5ra09344a)
Supplement: RA-016-D5RA09344A-s001 [file RA-016-D5RA09344A-s001.pdf]

Supplementary Information (SI) for RSC Advances.
This journal is © The Royal Society of Chemistry 2026

Supporting Information

Self-powered cathodic detection of dissolved oxygen using a paper-based biofuel cell

Isao Shitanda,[a,b,*] Riko Ohkura,[a] Noya Loew,[a], Hikari Watanabe,[a] Seiya Tsujimura,[b,c] and Masayuki Itagaki[a,b]

[a] Department of Pure and Applied Chemistry, Faculty of Science and Technology, Tokyo University of Science Noda, 2641 Yamazaki, Chiba 278-8510, Japan.

[b] Research Institute for Science and Technology, Tokyo University of Science, 2641 Yamazaki, Noda, Chiba 278-8510, Japan

[c] Division of Materials Sciences, Faculty of Pure and Applied Sciences, University of Tsukuba, 1-1-1 Tennodai, Tsukuba, Ibaraki, 305-8573, Japan

[*] Corresponding author: shitanda@rs.tus.ac.jp

For Special Issue on Development of electrochemical measurement techniques and devices to understand biological functions

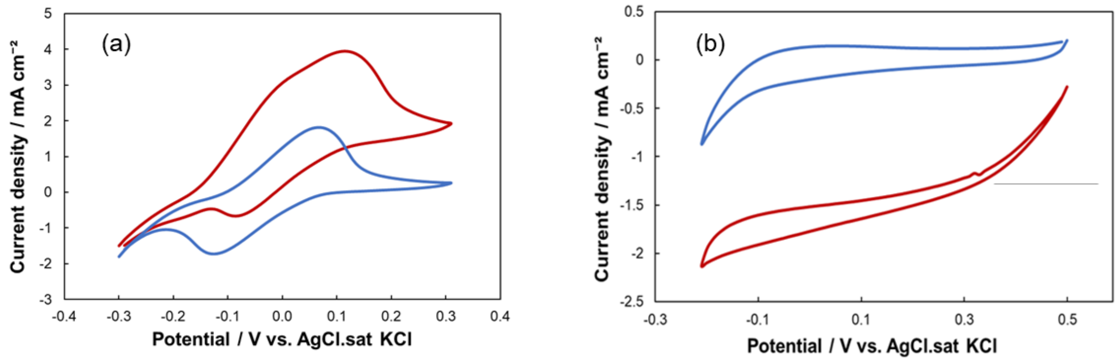

Figure S1. Cyclic voltammograms of the (a) anode and (b) cathode (red) with and (blue) without (a) glucose and (b) oxygen.
